# eDNA metabarcoding as a promising conservation tool to monitor fish diversity in Beijing water systems compared with ground cages

**DOI:** 10.1038/s41598-022-15488-w

**Published:** 2022-06-30

**Authors:** Mei Shen, Nengwen Xiao, Ziyi Zhao, Ningning Guo, Zunlan Luo, Guang Sun, Junsheng Li

**Affiliations:** 1 Present Address: State Key Laboratory of Environmental Criteria and Risk Assessment, Chinese Research Academy of Environmental Sciences, 8 Dayangfang, Beijing, 100012 China; 2 Lanzhou University, Lanzhou, 730000 China

**Keywords:** Biodiversity, Freshwater ecology

## Abstract

Fish diversity, an important indicator of the health of aquatic ecosystems, is declining sharply due to water pollution, overfishing, climate change, and species invasion. For protecting fish diversity, effective surveying and monitoring are prerequisites. In this study, eDNA (environmental DNA) metabarcoding and ground cages were used to survey the fish diversity of the Chaobai and Beiyun Rivers in Beijing. Based on the two methods, we identified 40 species, belonging to 35 genera, 18 families, and six orders. The richness of fish identified by eDNA metabarcoding was significantly higher than that captured by ground cages in both rivers. The fish captured by the ground cage method were all recognized by eDNA metabarcoding, except *Squalidus wolterstorffi* and *Saurogobio dabryi*, which were captured only in ground cages. The correlation of relative abundance between the two methods was affected by the properties of the rivers, such as the flow rate. Fish caught by ground cage in the Beiyun River were identified by eDNA, but not in the Chaobai River. Our results also suggest that the Chaobai River has higher fish diversity than the Beiyun River and different community assemblage. In addition to differences in the natural properties of the focal rivers, the development of urbanization is also an important contributor to different community structures overserved. eDNA metabarcoding as a new survey tool has great application prospects, it provides certain theoretical data and methodological references for the protection and management of river fish diversity.

## Introduction

Fish species account for one-quarter of the world's vertebrates, and their diversity is an important indicator of aquatic ecosystem health^1^. The 2020 Living Planet Index (LPI) shows that 3741 monitored populations have declined by an average of 84%, equivalent to a 4% decline per year since 1970. Freshwater fish biodiversity is declining much faster than that in oceans or forests^2^, owing to various factors such as water pollution, overfishing, and species invasion^3,4^.

Environmental DNA (eDNA) is a mixture of DNA from different species, such as microorganisms, animals, and plants. It includes the intracellular DNA emitted by the organism, as well as the extracellular DNA released into the environment after cell death by lysis^5–7^. Environmental DNA technology is used to capture DNA from environmental samples, to preserve, extract, amplify, sequence, and classify it, and then determine the distribution of organisms in the sampled environment^8^. So far, eDNA has been used to monitor the status of endangered^9^ and invasive^10^ species, and to assess the biomass^11^ and diversity^1,12,13^ of aquatic organisms, especially fish. However, in studies on fish diversity using the eDNA method, the water body is generally the ocean or a single river or lake^14,15^. Fewer studies have investigated fish diversity in multiple rivers^16^ by eDNA and compared the community structure of different rivers in combination with urban development.

Beijing is located in the north of China and is located across of five water systems, which belonging to the Haihe River System. There is rich fish diversity in Beijing, with more than 80 historical species^17–19^. A total of 93 species were identified from a traditional survey of fish in and around Beijing^20^. However, many surveys were long time ago, and the survey-methods, such as sticky nets, hand-scattered nets, and obtaining specimens from fishermen, are traditional with destructive^18,19^. Therefore, using cost-effective, repeatable, rapid, and no-invasive methods for monitoring and assessing fish diversity are crucial for the timely and effective management and conservation of fish in Beijing. The high-throughput sequencing (HTS) combined with eDNA—eDNA metabarcoding—is increasingly used as a powerful tool to monitor and assess fish diversity^1,12,21,22^.

Beijing is one of the fastest growing urban areas in China^23^. The urbanization rate of Beijing increased from 54.9 to 86.6%, from 1978 to 2019. Urbanization inevitably affects fish habitats through a reduction in the water area, aggravated pollution and channel hardening^24,25^. Urbanization is an essential trigger of species extinction and homogenization^26^. Urbanization has been carried out unevenly in regional Beijing. According to *Beijing Ecology and Environment Statement 2020* and Xiao^27^, the Beiyun River located in the central plain, has the highest urbanization rate, and the worst water quality of all of the rivers within Beijing. In contrast, the Chaobai River is located in the northern mountainous region, with a lower urbanization rate, and has the best water quality in Beijing.

To compare fish diversity and composition of eDNA metabarcoding with ground cages, fish diversity was surveyed in the Chaobai and Beiyun Rivers at different sites in Beijing. This article focused on three research objectives: (1) to present the results of two methods of surveying fish composition, diversity, and relative in two water systems; (2) to compare fish diversity collected by ground cages and eDNA metabarcoding, and analyze the correlations between the results of the two methods for exploring the feasibility of eDNA for surveying fish diversity in Beijing; (3) to compare the diversity and community structure of the two river systems and analyze the reasons about the result.

## Methods

### Sampling sites

**Figure 1:**
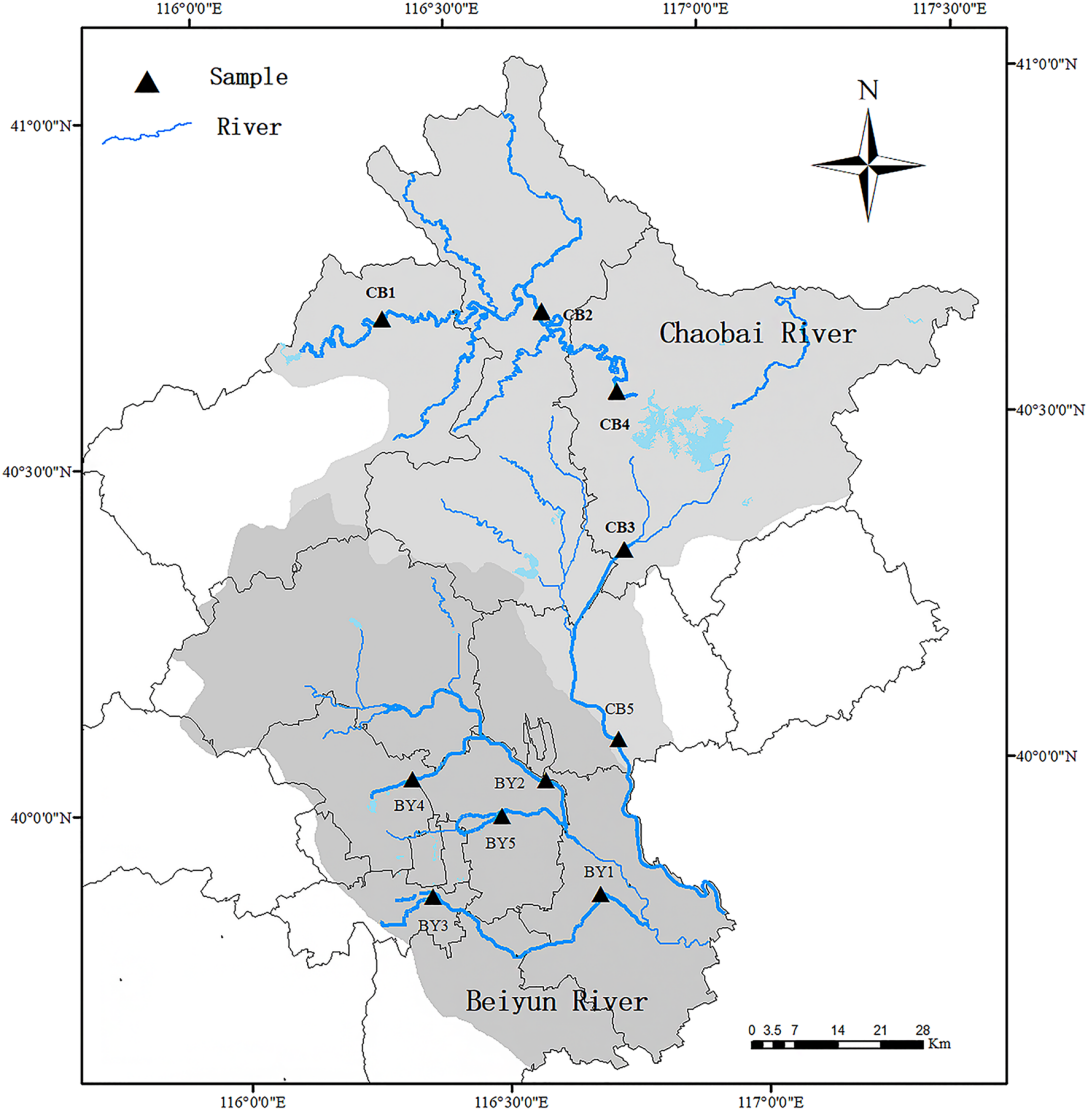
Sampling areas in Chaobai River ( light grey) and Beiyun River ( dark grey), with a total of 10 points. At each sampling point (▲), 3 L of water was used for eDNA while ground cages were used to capture fish. This map was created using Arcgis version 10.6 (https://desktop.arcgis.com/) based on the Resource and Environment Science and Data Center (https://www.resdc.cn/Default.aspx), edited by Mei Shen].

Five sites were surveyed in the Chao and Bai rivers, which belong to the Chaobai River. Addtional five sampling points were in the Beiyun, Wenyu, and Qinghe rivers, which are part of the Beiyun River. All water sampling was conducted in September 2020 (Fig. 1). The ground cage and eDNA metabarcoding methods were applied at each sampling point. All experiments were performed in accordance with *Laboratory animal—Guideline for ethical review of animal welfare*, ICS 65.020.30.

### Water samples collection

Surface water samples of 1 L were collected and mixed in a sterile, sealable brown plastic bottle and preserved at 4 °C for transporting to the laboratory. Three duplicate samples from one site were collected for eDNA analysis. Then, the samples were filtered through a 0.45 μm nylon filter membrane with a vacuum peristaltic pump. The glass funnel and filter head were thoroughly disinfected with 10% sodium hypochlorite before use. The filter membranes of each point were immediately placed into sterile 2 mL centrifugal tubes and stored at − 20 °C for the experiments. At each site, filtration blanks were established by filtering 1 L of distilled water in the same way to check for contamination during field collection.

### Capturing fish by ground cage

Ground cages were used as the traditional method to contrast with the eDNA metabarcoding. We followed the method of Xu^28^ with some modifications for considering various reasons and following the advice of fish experts, here is how we did it: three cages with a length of 2 m were placed at each site, and fish food was placed in the cages. The distance between the cages was about 3 m. The deployment time of the cages was 24 h to maximize the probability of detecting fish species. The water sample collection for eDNA, and fish acquisition by ground cages at each site were conducted simultaneously. This approach ensured that differences in fish assemblages determined between methods did not result from fish migration. After capturing, the fish specimens were stored on ice and transported to the laboratory within 10 h. To avoid potential bias in species assessment, identification was performed in a timely manner, and abundance at each site was recorded.

### DNA extraction and metabarcoding

The eDNA extraction experiment was completed at the Molecular Biology Laboratory of the Chinese Academy of Environmental Sciences, where all equipment are routinely decontaminated with UV light. The vessel were cleaned with 10% sodium hypochlorite and consumable material for extraction were autoclaved. DNA from the filters was extracted by Qiagen DNeasy Tissue and Blood DNA extraction kit (Qiagen, Venlo, the Netherlands) following the manufacturer's protocol with some modifications. The filter membrane obtained by 1 L water suction filtration was cut and placed in a 2 mL centrifuge tube. Then, 720 μL of ATL tissue lysis buffer reagent and 80 μL of proteinase K were added to the tube and mixed by shaking. Incubation of this mixture was performed at 56 °C for 3 h. Next, the samples were divided into two groups, and an 800 μL mixture of 400 μL AL lysis buffer & 400 μL anhydrous alcohol was added. Then, 500 μL of AW1 wash buffer and 500 μL of AW2 wash buffer were added to each tube and the tubes were centrifuged. Finally, 50 μL of AE buffer was added to each elution column, which was placed in a 37 °C water bath for 10 min before centrifuging. Filtration blanks and negative controls were co-extracted alongside the samples and were subjected to the same protocol. DNA was detected using 1.0% agarose gel. No data or bands were observed for the filtration blanks or negative controls.

Metabarcoding was performed in duplicate on each DNA extract with the primers MiFish-F (5′-GTCGGTAAAACTCGTGCCAGC-3′) and MiFish-R (5′-CATAGTGGGGTATCTA ATCCCAGTTTG-3′)^29^, which target the 12S rDNA region (~ 180 bp) of the mitochondrial genome, to identify fish species. Three PCR replicates were performed for each sample, the negative control and filtrate blanks. A two-step PCR protocol was adopted in this study. To determine the volume of DNA that should be added to the PCR reaction, using a Qubit 3.0 DNA Assay Kit for precise quantification of genomic DNA prior to the first round of PCR. The PCR assay volume was 30 μL and included 15 μL 2 × Hieff® Robust PCR Master Mix, 1 μL of the 10 μm forward and reverse primers, and 10–20 g of DNA template; ddH_2_O was added to 30 μL. For all samples, the first-step PCR was performed as follows: 94 °C for 2 min, followed by 30 cycles of 98 °C for 5 s, 50 °C for 10 s, and 72 °C for 10 s, with a final extension at 72 °C for 5 min. In the second-step PCR, Illumina bridge PCR-compatible primers were introduced into the sterile PCR tube (200 μL) to prepare the same reaction system as in the first-step PCR. The reaction solution was gently blown or stirred by pipette and briefly centrifuged to the bottom of the tube. The PCR procedure was as follows: 95 °C for 3 min, followed by 5 cycles of 94 °C for 20 s, 55 °C for 20 s, and 72 °C for 30 s, with a final extension at 72 °C for 5 min. After the two-step PCR amplification, the PCR products were detected using 1.0% agarose gel. None of the filtration blanks or negative controls exhibited amplification.

### High-throughput sequencing and bioinformatics analysis

The PCR products were utilized to construct libraries, followed by high-throughput sequencing on an Illumina MiSeq® using a MiSeq Reagent Kit v3 (600-cycle) (Sangon Biotech (Shanghai) Co., Ltd.). Paired-end sequence data (PE reads) were obtained by utilizing PEAR (v 0.9.8) to combine with tags based on overlaps^30^. Then, the samples were identified and distinguished according to the barcode tag sequence. To obtain clean reads, the data were filtered by Fastp (v 0.21.0)^31^ to eliminate adapter contamination and reads with low quality. USEARCH (v 11.0.667)^32^ was used to perform OTU (operational taxonomic unit) clustering of the optimized sequences of each sample according to 97% similarity, and chimeras were filtered out. All optimized sequences were mapped to each representative OTU sequence using USEARCH to obtain an OTU abundance table. Recently, ASV (Amplicon Sequence Variant) methods have been developed in the field of microbiology, replacing OTU methods due to their more accurate estimation of biological sequences^33^. But this study still used “clustering” of OTU rather than “denoising” of ASV.

The taxonomic assignment of OTU sequences was mapped to the fish gene database downloaded from the Mitochondrial Genome Database of Fish (MitoFish)^34^ website (http://mitofish.aori.u-tokyo.ac.jp/download.html) using the Blastn tool, all of Beijing's historical species are included in the database that we searched. Sequences were designated as belonging to a species if there was > 99% sequence identity to the MitoFish database barcode across the entire length of the amplicon, if a sequence from at least one other species within the same genus was available for comparison (and < 99% identical), and if the distribution of the species matched the published records of Beijing^20,35,36^. An OTU was categorized into another OTU if a sequence could not be assigned to a species. If a sequence could be assigned to several species (< 99% matching rate) and the species belonged to the same genus, the taxonomic resolution was collapsed to the genus level. Finally, OTUs representing each taxon were matched using TaxonKit (v 0.8.0.)^37^.

The eDNA data were compared with the data obtained by ground cages for the species richness table, and species and sequence abundance data were standardized to the proportions of the total sample. The relative abundance of each taxonomic rank was calculated from the OTU count table and the species count table. The vegan package (https://rdrr.io/cran/vegan/man/vegan-package.html) of R (v4.1.0) (https://www.r-project.org/ ) was used to calculate the fish richness of each sample, and a t-test was used to determine whether there were significant differences between methods. The beta diversity of the two water systems was sorted by non-metric multidimensional scaling (NMDS) based on the Bray–Curtis to determine the differences in species distribution between the two water systems.

## Results

### Fish diversity

**Figure 2:**
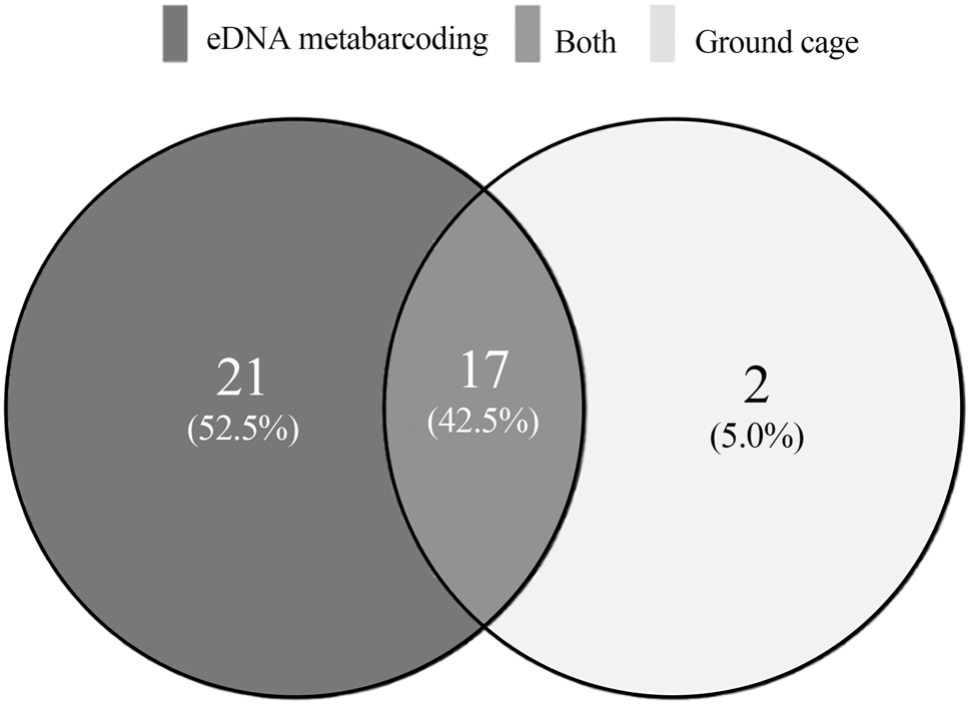
Number of fish species identified by eDNA (eDNA metabarcoding) metabarcoding and ground cages (17 taxa were detected by both methods, 21 taxa were detected only by eDNA, and two taxa were detected only by ground cages).

Based on eDNA metabarcoding 50 fish OTUs, resulting in 38 fish species were identified from 30 sampling events (33 genera, 18 families, six orders), whereas from ground cages 19 species from a total of 711 individuals were identified (19 genera, six families, and two orders). Fish species obtained by the two methods comprised 40 species in 35 genera, 18 families, and six orders (Table 1). Compared with the ground cage results, higher numbers of species, genera, families, and orders were detected by eDNA metabarcoding. In total, 17 species were detected by both eDNA metabarcoding and ground cages, and 21 species were identified by eDNA metabarcoding alone. Squalidus wolterstorffi and Saurogobio dabryi were caught by ground cages exclusively (Fig. 2).

**Table 1:** Sequence numbers of fish detected by eDNA and caught by ground cages at 10 sites in Beijing.

Order	Species	eDNA (sequence)			Traditional (Number)		
		Chaobai	Beiyun	Total	Chaobai	Beiyun	Total
Beloniformes	*Oryzias latipes*	1073	3008	4081	0	0	0
Cypriniformes	*Abbottina rivularis*	924	2301	3225	19	6	25
	*Acheilognathus macropterus*	46	874	920	4	2	6
	*Carassius auratus*	26,614	165,549	192,163	0	0	0
	*Chanodichthys erythropterus*	84	39	123	3	0	3
	*Cobitis melanoleuca*	1726	0	1726	0	0	0
	*Cyprinus carpio*	9189	3151	12,340	0	0	0
	*Gnathopogon strigatus*	140	0	140	0	0	0
	*Hypophthalmichthys molitrix*	640	0	640	0	0	0
	*Squalidus wolterstorffi*	0	0	0	5	0	5
	*Hypophthalmichthys nobilis*	21,193	357	21,550	0	0	0
	*Misgurnus anguillicaudatus*	762	42,882	43,644	4	15	19
	*Cobitis granoci*	1393	2415	3808	1	0	1
	*Saurogobio dabryi*	0	0	0	4	0	0
	*Opsariichthys uncirostris*	2440	39	2479	1	0	1
	*Paramisgurnus dabryanus*	3399	643	4042	99	3	102
	*Pseudorasbora parva*	5265	42,561	47,826	36	158	194
	*Rhodeus ocellatus*	758	14,452	15,210	1	17	18
	*Rhynchocypris lagowskii*	24,493	177	24,670	11	0	11
	*Sarcocheilichthys soldatovi*	2890	393	3283	5	0	5
	*Ctenopharyngodon idella*	299	332	631	0	0	0
	*Zacco platypus*	31,034	1243	32,277	0	0	0
	*Hemiculter leucisculus*	618	1145	1763	289	0	289
Perciformes	*Micropterus salmoides*	0	219	219	0	0	0
	*Rhinogobius cliffordpopei*	0	176	176	0	0	0
	*Channa argus*	4962	20,092	25,054	2	0	2
	*Lateolabrax maculatus*	0	32	32	0	0	0
	*Macropodus chinensis*	366	505	871	5	0	5
	*Micropercops swinhonis*	7263	61,546	68,809	9	0	9
	*Odontobutis potamophila*	90	5620	5710	0	8	0
	*Rhinogobius brunneus*	1365	424	1789	3	1	4
	*Rhinogobius similis*	3012	1037	4049	0	0	0
	*Siniperca chuatsi*	770	204	2773	0	0	0
Salmoniformes	*Hypomesus olidus*	260	0	260	0	0	0
	*Oncorhynchus mykiss*	1647	0	1647	0	0	0
Siluriformes	*Ictalurus punctatus*	0	85	85	0	0	0
	*Silurus asotus*	6842	514	7356	0	0	0
	*Tachysurus fulvidraco*	3552	3871	7423	0	0	0
	*Tachysurus ussuriensis*	1393	32	1425	0	0	0
Synbranchiformes	*Monopterus albus*	259	136	395	0	0	0

**Figure 3:**
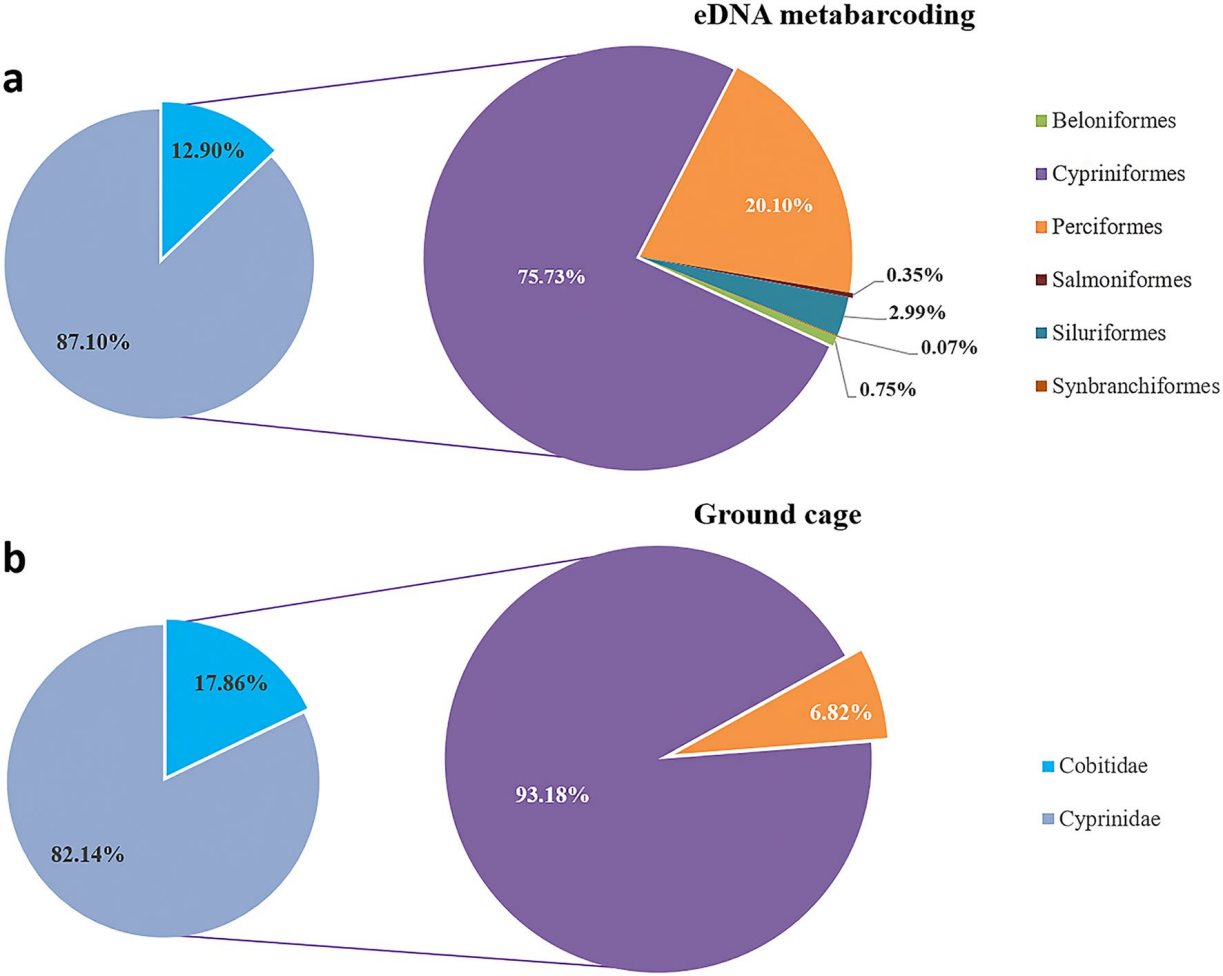
Relative abundance of fish at the order level detected by the two methods and relative abundance of occurrence at the family level corresponding to Cypriniformes.

Six orders were detected by eDNA metabarcoding. Cypriniformes and Perciformes were the dominant orders, with a sequence abundance in the total sequence abundance of 75.73% and 20.10% respectively. Siluriformes was the most common order, accounting for 2.99% of all sequences. Beloniformes (0.75%), Salmoniformes (0.35%), and Synbranchiformes (0.07%) were rare orders. The dominant families in Cypriniformes were Cyprinidae (87.1%) and Cobitidae (12.90%) (Fig. 3a). The less abundant orders contained only 1–2 species, such as *Oryzias latipes* in Beloniformes, *Hypomesus olidus* and *Oncorhynchus mykiss* in Salmoniformes, and *Monopterus albus* in Synbranchiformes (Table 1). Only Cypriniformes (93.18%) and Perciformes (6.82%) were captured by the ground cage method. Cypriniformes was also composed of Cyprinidae (82.14%) and Cobitidae (17.86%) (Fig. 3b). Among them, the dominant species were *Hemiculter leucisculus* and *Pseudorasbora parva* in Cyprinidae and *Paramisgurnus dabryanus* in Cobitidae (Table 1).

### Comparison and relationship between eDNA metabarcoding and ground cages

**Figure 4:**
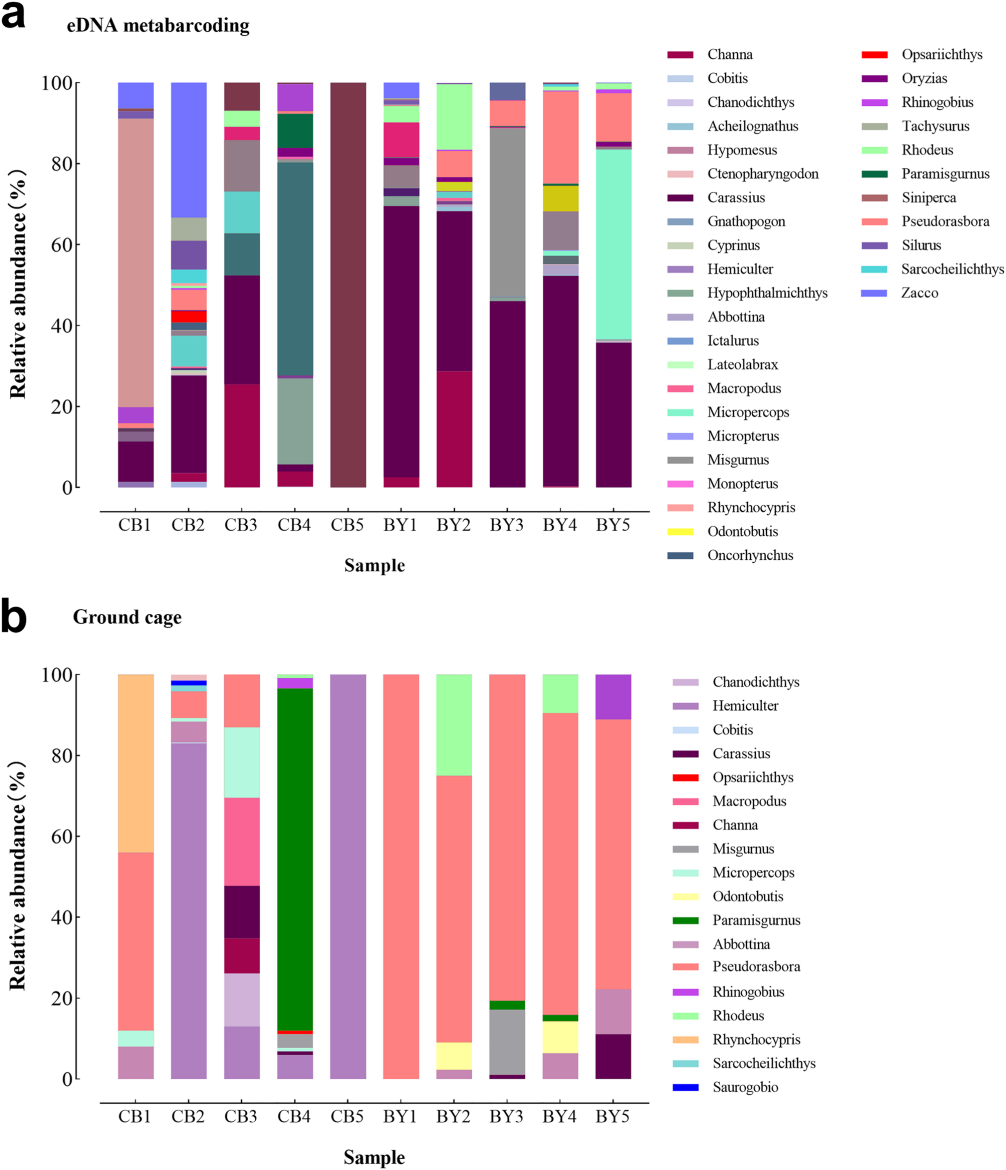
Relative abundance (%) of fish detected at each sampling point at the genus level by (**a**) eDNA metabarcoding and (**b**) ground cages.

At the genus level, the diversity obtained by eDNA metabarcoding (n = 33) was higher than that obtained by ground cages (n = 19). The genera with high relative abundance based on eDNA metabarcoding were *Carassius* (35.28%), followed by *Micropercops* (12.63%) (Fig. 4). The genera with high relative abundance captured by ground cages were *Hemiculter* (40.65%) and *Pseudorasbora* (27.28%) (Fig. 4).

**Figure 5:**
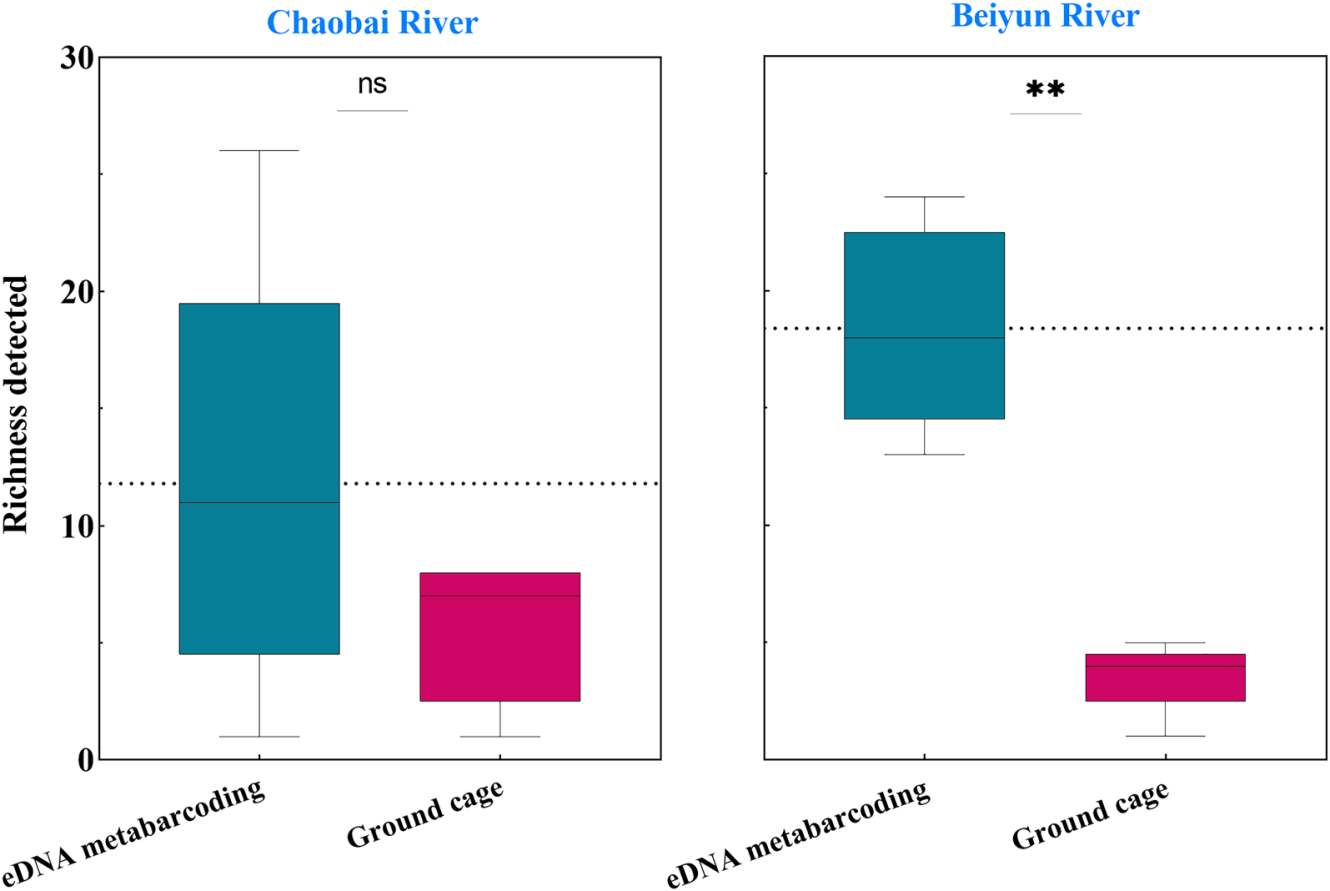
Richness detected at each site using eDNA metabarcoding (red) or ground cage fishing (blue). Differences between the methods were determined using t-tests (number of species followed a normal distribution); Chaobai River P > 0.05; Beiyun River P < 0.05.

The fish richness at each site detected by eDNA metabarcoding (average = 15.10 (SD = 7.19)) was significantly higher than that captured by ground (average = 4.60 (SD = 2.37); Welch two-sample t-test, T = 4.1603, df = 10.941, p < 0.05). The fish richness identified using eDNA metabarcoding in the Chaobai River (n = 34) and the Beiyun River (n = 18) was higher than that identified with ground cages (Fig. 5). The difference was significant of the Beiyun River (Welch two-sample t-test, t = 1.93, df = 4.00, p < 0.05; Fig. 5) but not significant with the Chaobai River (Welch two-sample t-test, t = 1.93, df = 4.00, p = 0.13).

**Figure 6:**
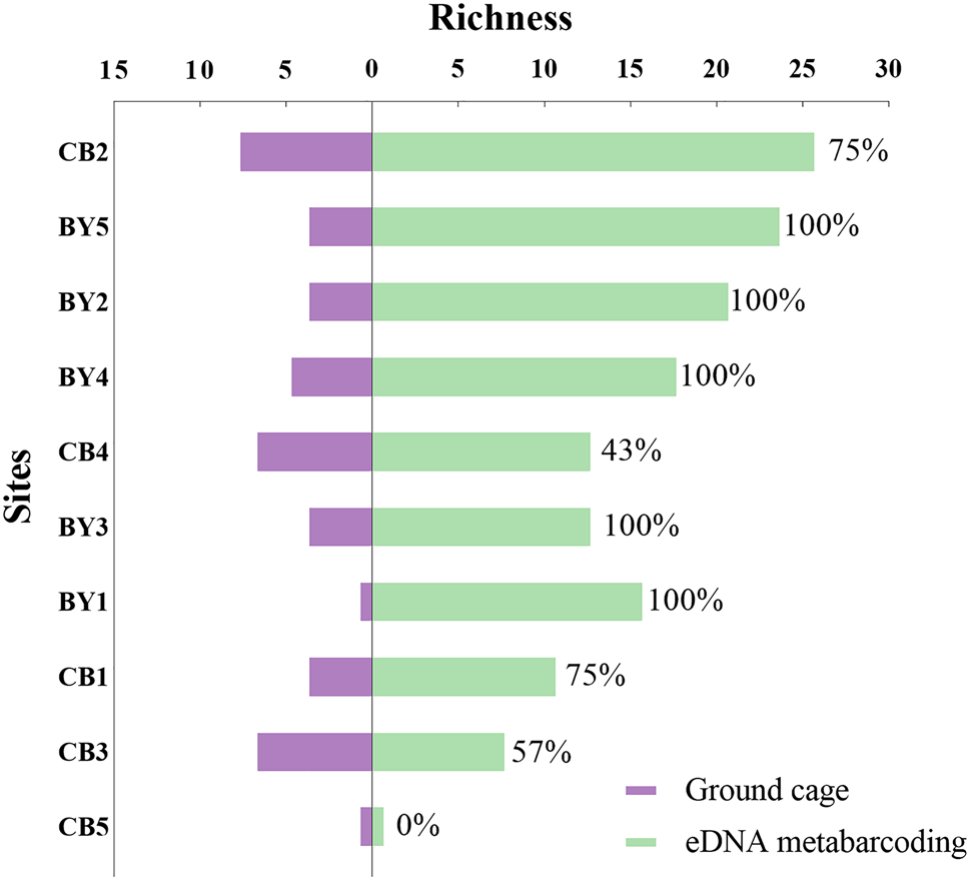
Species richness captured by both methods (the % is the proportion of the number of species obtained by both methods to species richness captured by ground cage alone).

The fish richness obtained by the two methods together at each site as a proportion of the fish richness captured by the ground cage method only is used to illustrate the capture efficiency of the eDNA metabarcoding, expressed as a percentage in the figure (Fig. 6). The abundance of fish identified by the eDNA metabarcoding is higher or equal to that of the traditional method. The fish obtained by both methods at all points in the Beiyun River accounted for 100% of the fish caught by the traditional method alone. However, the percentages of all sites in Chaobai River is less than 100% (Fig. 6). Although both the eDNA metabarcoding and the ground cage method captured one species at CB5, but the species is not the same species. *Siniperca chuatsi* was detected by eDNA metabarcoding, but the species was captured by ground cage is *Hemiculter leucisculus.*

### Diversity of the Chaobai and Beiyun Rivers

The fish richness was slightly higher in the Chaobai River (36 species: 34 based on eDNA metabarcoding, 18 based on ground cages) than in the Beiyun River (33 species: 33 based on eDNA metabarcoding, 8 based on ground cages) (Table 1). For the species detected by eDNA, the most abundant in the Chaobai River was *Zacco platypus* (18.41%), followed by *Carassius auratus* (15.79%), *Rhynchocypris lagowskii* (14.53%), and *Hypophthalmichthys nobilis* (12.57%). *Carassius auratus* (44.02%) was the most abundant species detected in the Beiyun River, followed by *Micropercops swinhonis* (16.37%), *Misgurnus anguillicaudatus* (11.40%), and *Pseudorasbora parva* (11.32%) (Table 1).

The distribution of the top 10 most abundant species in the two rivers shown that the most abundant species caught by ground cages in the Chaobai River was *Hemiculter leucisculus* (57.80%), followed by *Paramisgurnus dabryanus* (19.80%), *Pseudorasbora parva* (7.20%), and *Abbottina rivularis* (3.80%). The most abundant species in the Beiyun River was *Pseudorasbora parva* (75.24%), followed by *Rhodeus ocellatus* (8.09%), *Misgurnus anguillicaudatus* (7.14%), and *Odontobutis potamophila* (3.81%) (Table 1).

**Figure 7:**
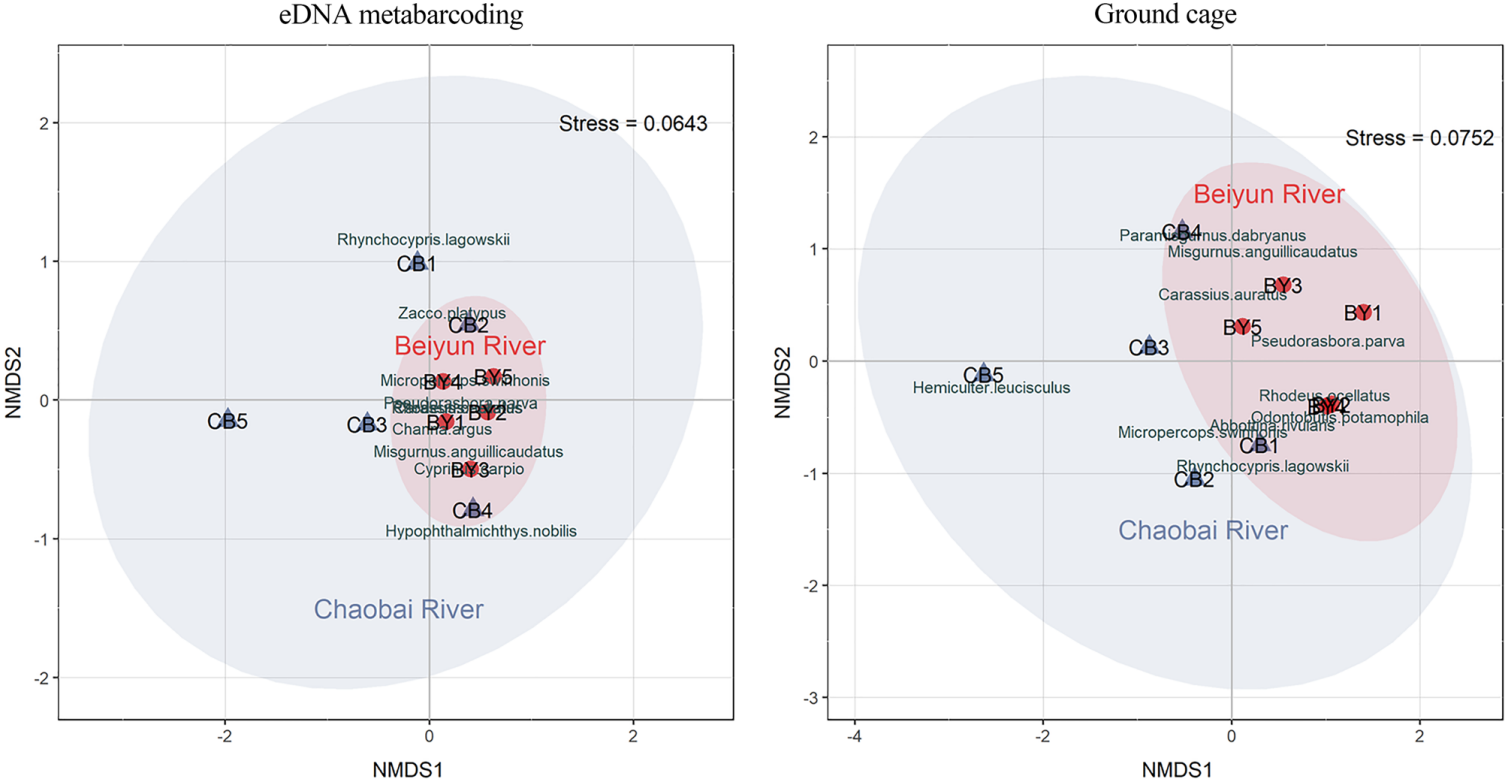
Community variation based on Bray–Curtis dissimilar non-metric multiscale (NMDS) sorting: the left panel is based on the results of eDNA metabarcoding; the right panel is based on ground cage data.

Beta diversity showed a separation of the fish communities between the Chaobai and Beiyun Rivers by both the eDNA metabarcoding and ground cage method (Fig. 7). However, the distribution of samples in the Beiyun River was more concentrated, while samples in the Chaobai River were more scattered, and the distance between CB5 and the other sample points was very far.

## Discussion

Fish are an important component of aquatic ecosystems and have a significant impact on the balance of an ecosystem. Fish can also be used as an indicator to evaluate the biological integrity of water bodies^38^. The application of nondestructive techniques to investigate fish diversity is becoming an inevitable trend. In some studies on fish diversity in Beijing^20,36^, the results showed a gradual decline of fish diversity due to the influence of human activities. In this study, we demonstrated proof that eDNA can be used for monitoring fish diversity in river, as the species richness detected by the eDNA metabarcoding was higher than captured traditional method, supporting other studies investigating riverine fishes^16^. We also showed that the fish community structure of Beiyun River that is highly urbanized is relatively homogeneous compared with Chaobai River that occurs in a montane environment.

### Fish diversity

The 40 fish species detected in this study (Table 1) were recorded in the historical survey conducted in Beijing^20,36^. Dominant groups belonged to Cypriniformes, Cyprinidae, and Cobiaceae (Fig. 3). Our result is consistent with that of Zhang, who showed that the relative abundance of Cypriniformes, Perciformes, Cyprinidae and Cobitidae was 72.0%, 11.8%, 68.0% and 12.0%, respectively^20^. As such our study indicated that the dominant fish species in Beijing have not changed in the past decades.

There were 17 fish species obtained by both methods (Fig. 2). Among them, *Pseudorasbora parva*, *Abbottina rivularis*, *Hemiculter leucisculus*, *Paramisgurnus dabryanus*, *Rhodeus ocellatus*, *Carassius auratus*, *Opsariichthys uncirostris*, and *Micropercops swinhonis* are common species in eastern China and are the dominant species investigated in research^20,39^. *Cobitis granoci* and *Rhynchocypris lagowskii* have adapted to the cold climate in the north^39^. However, it is worth noting that *Odontobutis potamophila* is generally distributed in the middle and lower reaches of the Yangtze River^40^. This species was not recorded in the historical survey results of *Beijing Fishes, Amphibians and Reptiles* (1994) until recently^36,41^. Such fish may have been introduced due to south-to-north water diversion or may have been released by citizens. *Carassius auratus*, *Opsariichthys uncirostris*, and other fish are economic species. However, there are some small non-economic fish species, such as *Pseudorasbora parva*, *Abbottina rivularis*, *Macropodus chinensis*, that play an important role in the ecosystem and are an important food source for some birds^19^.

### Richness differences between Rivers

The results of the two methods showed that the fish abundance in the Chaobai River was higher than that in the Beiyun River (Table 1), and this was also shown in the study conducted by Du^36^. Rapid urbanization leads to human activities increase, river pollution, and channel hardening, which have all contributed to the decline in the quality of fish’s natural habitat^20,24,42^. The ecological health of the water is generally worse in the Beiyun River in the center of Beijing city^43^ compared with the Chaobai River with less human interference.

Obvious spatial differences in the fish community structure of the two rivers were also found. Apart from the interference of human activities, there is also the influence of natural factors such as water temperature and altitude^44,45^. Differences in habitats lead to differences in community structure^46,47^. The Beiyun River is dominated by *Pseudorasbora parva*, *Misgurnus anguillicaudatus*, and *Carassius auratus*, who are low-altitude pollution-tolerant species; while the Chaobai River is dominated by *Zacco platypus*, *Rhynchocypris lagowskii*, and *Opsariichthys uncirostris*, whose habitat is in high-altitude and low-temperature, this is consistent with the results of study Zhang and Du^20,36^. Moreover, the fish abundance of the samples in the Beiyun River was relatively uniform, and the difference in fish abundance among the samples in the Chaobai River was relatively large (Fig. 7). It may be that the sample sites of the Chaobai River span a larger distance and the spatial heterogeneity is more prominent, resulting in obvious differences in diversity between sampling points. Therefore, eDNA technology is able to distinguish the composition of fish communities in different habitats.

### eDNA metabarcoding as a transformative tool for assessing fish diversity in rivers of Beijing

Most of the traditional methods that have been used are harmful to fish, such as ultrasonic fish devices, hanging nets, and hand-cast nets^19,36,39^. Many studies^21,48–50^ have shown that eDNA metabarcoding for monitoring fish diversity is feasible and efficient, and the result of eDNA metabarcoding are superior to traditional methods^1,12^. In the present study, eDNA metabarcoding identified more fish species than ground cages (Table 1, Figs. 3, 4) in both of the water systems (Fig. 6). eDNA can not only detect conventional species but can also easily identify species with low abundance. For example, *Hypomesus olidus*, *Chanodichthys erythropterus*, and other fish species were detected by eDNA metabarcoding only. eDNA has been used for the detection of endangered and rare species^51–53^. Although eDNA failed to identify all fish species (such as *Squalidus wolterstorffi* and *Saurogobio dabryi* in this study), it may be insufficient water samples were collected and without stratified sampling, although the database contains all of Beijing's historical fish species. However, the high detection rate of eDNA makes it well positioned to become a mainstream monitoring method, and it also overcomes the limitations of traditional survey methods and reduces fish pressure and habitat destruction.

Many studies have compared results of eDNA with those from traditional methods and have suggested a correlation between them^54,55^, but the specific relationship is not currently clear. Because different stages are affecting the state of eDNA, such as the origin of eDNA, degradation and decay of eDNA, transport of eDNA, and the microbiome datasets generated by HTS are compositional because the datasets have an arbitrary total imposed by the instrument^56,57^. The relationship between absolute abundance in the environment and the relative abundance after sequencing is not predictable. PCR cycle number and primer amplification efficiency also effects the results of diversity metrics in sequencing studies^56–58^. In our study, we analysis of metabarcoding datasets assume that sequencing data are equivalent to ecological data, of course, there are some limitations to this.

The fish caught by ground cage at each site in the Beiyun River were all detected by eDNA metabarcoding. But some fish were caught by ground cage but not detected by eDNA metabarcoding of sites in the Chaobai River. We can infer that this was determined by the characteristics of the river. Compared with lakes and reservoirs, rivers have temporal and spatial variability^14,59^. In addition, DNA can persist for several days in water^60^, so the species detected by eDNA metabarcoding may not originate from this habitat (the upper stream)^22,61,62^. The Chaobai is a mountainous river with high flow velocity. on the contrary, the Beiyun River locate in the urban with low velocity . The results detected by eDNA basically originated from the sampling points themselves and were highly correlated with result of traditional methods. Therefore, in the investigation of river fish diversity by eDNA metabarcoding, the characteristics of the river should also be considered, especially in the study of some river segments. Of course it cannot be excluded that the traditional method capture fewer fish in the Beiyun River.

## Conclusions

eDNA metabarcoding is a developing trend in aquatic biodiversity investigation because of its wide recognition ability. This study used eDNA metabarcoding and ground cages to investigate fish diversity in two river systems in Beijing. A total of 40 fish species were surveyed at 10 sites, and eDNA metabarcoding identified 38 species. The results showed that compared with the ground cage method, eDNA metabarcoding recorded higher fish abundance. The correlation between the the result of eDNA and ground cage was different at each sites of the two water systems. These findings shown the feasibility of eDNA metabarcoding for the investigation of fish diversity in Beijing rivers and provide new reference data for the management and protection of Beijing river fish. The survey results also suggest that the diversity in mountain rivers is higher than that in lowland urban river. The fish community structures of the two rivers were also different. Although eDNA metabarcoding cannot identify the biological characteristics of organisms, it is an important tool for aquatic biodiversity monitoring.

## Data Availability

The datasets generated during the current study are available in the NCBI repository, Accession number is PRJNA842175.
